# How social representations of sexually transmitted infections influence experiences of genito-urinary symptoms and care-seeking in Britain: mixed methods study protocol

**DOI:** 10.1186/s12889-016-3261-0

**Published:** 2016-07-11

**Authors:** Fiona Mapp, Ford Hickson, Catherine H. Mercer, Kaye Wellings

**Affiliations:** Department of Social and Environmental Health Research, Faculty of Public Health and Policy, London School of Hygiene & Tropical Medicine, London, UK; Centre for Sexual Health and HIV Research, Research Department of Infection and Population Health, University College London, Mortimer Market Centre, London, UK

**Keywords:** Mixed methods research, Natsal-3, Survey, Semi-structured interviews, Sexually transmitted infections, Sexually transmitted disease, Genito-urinary symptoms, Care-seeking behaviour, Non-attendance

## Abstract

**Background:**

Social understandings of sexually transmitted infections and associated symptoms and care-seeking behaviour continue to lag behind advancements in biomedical diagnostics and treatment, perpetuating the burden of disease. There is a lack of research linking perceptions, experiences and care-seeking for sexual health issues, especially research conducted outside of medical settings. We aim to explore lay perceptions of STIs and how these influence experiences of genito-urinary symptoms and associated care-seeking behaviour, in women and men in Britain.

**Methods and design:**

This study adopts a participant-selection variant of the explanatory sequential mixed methods design to incorporate quantitative and qualitative strands. We use data from Britain’s third National Survey of Sexual Attitudes and Lifestyles (*n* = 15,162) to analyse national patterns of symptom experience and care-seeking, and to identify a purposive qualitative sample. Semi-structured interviews (*n* = 27) following up with survey participants include a novel flash card activity providing qualitative data about infection perceptions, symptom experiences and decisions about healthcare. Quantitative and qualitative data are analysed separately using complex survey analyses and principles of Interpretative Phenomenological Analysis respectively. Data are then integrated in a subsequent phase of analysis using matrices to compare, contrast and identify silences from each method.

**Discussion:**

This is an ongoing mixed methods study collecting, analysing and synthesising linked data from a national survey and follow-up semi-structured interviews. It adds explanatory potential to existing national survey data and is likely to inform future surveys about sexual health. Given the current uncertainty around service provision in Britain, this study provides timely data about symptom experiences and care-seeking behaviour which may inform future commissioning of sexual healthcare.

## Background

### Sexually transmitted infections

Sexually transmitted infections (STIs) remain a persistent public health challenge across populations, despite advancements in diagnostics and treatment options. In England, STIs disproportionately affect people under 25 years and men who have sex with men [1], although the rate of STIs in older people is increasing [2]. So far progress in the social understanding and mitigation of negative perceptions of STIs has lagged behind biomedical advancements. Stigma has been pronounced as the greatest barrier to healthcare-seeking in relation to STI care [3] resulting in increased morbidity and mortality and repercussions at an individual, health system and policy level. Despite some progress in elucidating sources and outcomes of stigma [4], the underlying mechanisms through which stigma influences individual experiences are not sufficiently explained in relation to STIs.

### Genito-urinary symptoms

Genito-urinary symptoms are commonly associated with STIs but can also be caused by other urinary tract infections [5, 6] and cancers [7]. There are currently no estimates of how common genito-urinary symptoms are among the general population, who they affect, or how individuals interpret and respond to them. Generally symptoms are unpleasant, subjective experiences that alert individuals to a change within their body [8]. Experiencing sensations or symptoms does not always trigger care-seeking if symptoms are not interpreted as a need for care [9, 10]. The overlapping symptomatology emphasises the need to better understand experiences, meanings and decision-making about symptoms, underlying causes and care needs and Scott and Walter [9] advocate symptom level research rather than disease level research to better understand symptoms in relation to care-seeking.

### Care-seeking behaviour

Seeking healthcare is a complex and heterogeneous behaviour and decisions about individual health needs are based on diverse motivations and information sources. Fortenberry’s work on adolescent sexually transmitted disease care offers a definition of healthcare-seeking as the *“interval between recognition of a health problem and its clinical resolution and… the accompanying cognitive and behavioural responses”* ([11] p147). This incorporates a temporal dimension to care-seeking which is important in terms of early detection, diagnosis and treatment of contagious pathogens. This definition however, excludes the earlier process of sense-making that occurs before recognising a change in health and also neglects what happens if individuals do not attend a medical service.

In the UK, specialist sexual health clinics (also known as STI clinics and genito-urinary (GUM) clinics) are open access and free at the point of care. They are the best equipped service to diagnose and manage a range of genito-urinary conditions, providing more comprehensive STI screenings than community services and diagnosing and treating more STIs [12]. However some people find specialised clinics stigmatizing and unfamiliar environments [13] and so primary care is also a vital part of genito-urinary healthcare [14, 15].

We need to understand the experiences, priorities and decisions of those in need of healthcare outside of medical settings if we are to improve pathways in to care.

### Rationale for this study

STIs, symptoms and care-seeking are topics which lack in-depth social examination, having been dominated by biomedically-framed research. It is unclear to what extent lay perceptions and social representations of STIs influence conceptualisations and experiences of genito-urinary symptoms, and what determines care-seeking behaviours for symptoms of STIs. Care-seeking has already been described as a complex research topic [9, 16] and there are additional complexities associated with genital symptoms and associations with stigmatised conditions such as STIs. Therefore a mixed methods approach is needed to incorporate complexities of the research topic through mixing types of methods and types of data.

There are many ways of defining mixed methods research depending on the methodological and philosophical approach. In this study we use the following core principles of mixed methods research [17] to guide our study from conception to completion:

- Collecting and analysing both qualitative and quantitative data in a single study
- Integrating the different forms of data after separate quantitative and qualitative analyses
- Study design determined by the research questions [18]
- Priority given to explanatory qualitative data
- Study is theoretically grounded drawing on pragmatism
- Using mixed methods for *“the broad pursuit of breadth and depth of understanding”* ([19] p123)

Mixed methods helps transcend single dimension and linear understandings of the topic and produce multi-dimensional insights [20] into symptom experiences and care-seeking whilst offsetting weaknesses of quantitative and qualitative methods. Our research questions necessitate different mixed methods reasoning to produce appropriate data including: data complementarity to illustrate findings from the other method; development of one method from another; expansion to examine different aspects of the same phenomenon; and some triangulation to corroborate findings where there is sufficient overlap of data [21].

### Aim and research questions

This study aims to explore lay perceptions of STIs and how these influence experience of genito-urinary symptoms and associated care-seeking behaviour, focussing on non-attendance in women and men in Britain. The main research questions are:

1. What are the social representations of STIs?
2. How does stigma influence experiences of genito-urinary symptoms and care-seeking?
3. How do people interpret genito-urinary symptoms?
4. Why do some people with genito-urinary symptoms not seek care at sexual health clinics?

Our research is framed by, although not restricted to, sexual health.

## Methods and design

### Study design

**Fig. 1:**
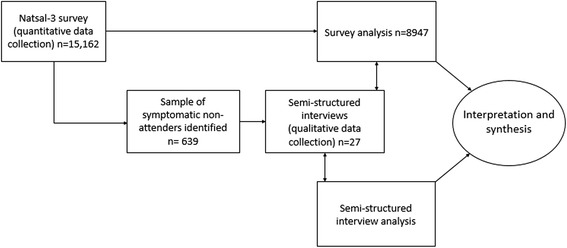
Mixed methods study design

We use a participant-selection variant of the explanatory sequential mixed methods design (Fig. 1) [18, 22]. Data collection takes place in two distinct stages to enable us to use the quantitative survey data from the third National Survey of Sexual Attitudes and Lifestyles (Natsal-3) which is collected first, to identify the sampling frame for the dominant qualitative strand giving us linked datasets. Analyses of the quantitative and qualitative strands are conducted independently but simultaneously to maintain the integrity of each data. Key findings from each strand are integrated in a second stage of analysis to produce synergistic interpretations about genito-urinary symptoms and care-seeking behaviour and deepen understanding of the research topic. Our sequential design enables identification of a sample with a potential need for healthcare, outside of medical settings. The linked datasets increase explanatory and integrative potential of the data. The study is under-pinned by public health approaches to individual and population health needs, as well as sociological and psychological theory. We draw on principles of pragmatism to incorporate different research paradigms ([23] p26) ([24] p14–16) within the study and use phenomenology to focus on lived experiences ([25] p1–21).

### Study setting

Natsal-3 is conducted in Britain involving random population sampling of women and men based on household addresses. Follow-up qualitative interviews are carried out in England and Wales with a small sub-set of participants selected based on their survey responses.

### Quantitative strand

The quantitative strand comprises secondary analysis of Natsal-3 survey data.

### Natsal-3 survey data

The National Surveys of Sexual Attitudes and Lifestyles conducted over the past three decades have provided detailed information about sexual behaviour in Britain [26]. The third of these surveys, Natsal-3, was carried out between September 2010 and August 2012 interviewing 15,162 women and men in total. Full details of the methods have been reported elsewhere [27] but are summarised here so the survey can be understood in the context of this mixed methods study. Participants were aged 16–74 years (with over-sampling of people aged 16–34 years) and sampling took place across England, Scotland and Wales. A multi-stage, clustered and stratified probability sample design was used and data were weighted to represent the British population according to age, gender and geographic region. Weighting the data also accounted for differing selection and non-response probabilities. A combination of computer assisted personal interview (CAPI) and, for the more sensitive topics, computer assisted self-interview (CASI) was used. Overall the response rate to Natsal-3 was 57.7 % [27, 28].

There were five main domains of questions in the Natsal-3 questionnaire which included questions on health, family and learning about sex; first sexual experiences, use of contraception and sexual lifestyle; sexual behaviour including number of partners, sexual practices, sexual health and reproduction; attitudes and risks; and socio-demographic information. Urine samples were collected from a sub-sample of sexually-experienced participants aged 16–44 to test for *Chlamydia trachomatis*, * Neisseria gonorrhoeae*, *Mycoplasma genitalium*, Human Papillomavirus and HIV antibodies, with subsequent testing for *Trichomonas vaginalis* [29]. Another sub-sample of participants aged 16–74 provided saliva samples for testosterone measurement [27].

In the CASI, questions were asked about attendance at sexual health clinics and experience of genito-urinary symptoms in the month prior to data collection.

### Quantitative data analysis

**Fig. 2:**
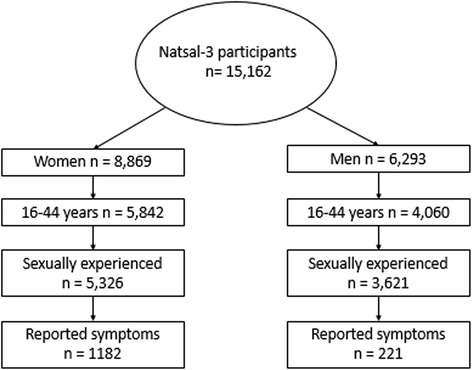
Derivation of sub-groups for quantitative analyses

This study uses data from a sub-sample of Natsal-3 participants (Fig. 2) who were aged 16–44 years and sexually experienced, defined as those who reported having had at least one sexual partner (*n* = 8947). We want to capture and compare patterns of symptoms and care-seeking across a greater age range than those at highest risk. After initial data exploration involving cross-tabulations of key variables and basic summary statistics to facilitate choosing the sampling frame for the qualitative strand, data analysis was delayed to coincide with analysis of the qualitative data. This approach enables us to move between each dataset, using findings from one to inform analyses of the other, and vice versa, whilst maintaining analytical distinction between data types. We conduct statistical analyses on variables derived from the survey questions [30] *“In the last month, that is since (date one month ago), have you had any of the following symptoms?”* and *“Have you ever attended a sexual health clinic (GUM clinic)?”* The primary dependent variables are reported symptom experience and non-attendance at a sexual health clinic. Table 1 shows the symptoms that the Natsal-3 questions asked about. Independent variables included in the quantitative analyses are informed by qualitative findings and relevant literature. Participants with missing data for either the independent and/or the dependent variables are excluded from analysis as there are generally low levels of missing data in Natsal-3, often between 1 and 3 % [28]. We are using the survey commands in Stata V.14.1 to account for stratification, clustering and weighting of the dataset. Prevalence estimates of reported symptoms and non-attendance at sexual health clinics are calculated with 95 % confidence intervals for women and men, stratified by age-group. We are using logistic regression to examine associations between reporting symptoms and not having attended a sexual health clinic in the past year to produce crude and age-adjusted odds ratios. Analyses are stratified by sex to reflect differences in male and female anatomy, physiology and epidemiology of genito-urinary infections [29, 31, 32] and reported gender differences in care-seeking behaviour [33–35].

**Table 1:** Symptoms reported in Natsal-3 and follow-up qualitative interviews

Symptoms reported	Crude number of symptoms reported in Natsal-3 survey interview (past month)	Symptoms reported by qualitative participants during Natsal-3 interview (past month)	Symptoms reported by qualitative participants during semi-structured interview (ever)
Women			
Pain, burning or stinging when passing urine	384	2	14
Genital wart/lump	35	1	0
Genital ulcer/sore	25	3	1
Abnormal vaginal discharge	236	0	13
Unpleasant odour associated with vaginal discharge	206	4	5
Vaginal pain during sex	304	1	8
Abnormal bleeding between periods	245	4	6
Bleeding after sex (not during a period)	154	1	3
Lower abdominal or pelvic pain (not related to periods)	305	5	9
Men			
Pain, burning or stinging when passing urine	101	3	7
Genital wart/lump	27	1	2
Genital ulcer/sore	7	0	0
Discharge from the end of the penis	19	1	2
Painful testicles	93	7	8

(Two men reported no symptoms during their semi-structured interviews although they had indicated experiencing symptoms in their initial Natsal-3 interview)

### Qualitative strand

The qualitative strand comprises semi-structured interviews with Natsal-3 participants (Fig. 1).

### Sampling and data collection

We identified a sample of 639 Natsal-3 participants who had reported firstly, at least one genito-urinary symptom, secondly, no previously reported attendance at a clinic and thirdly consent to be re-contacted. From the Natsal-3 data, 79.9 % (95 % CI 76.0–83.3) of participants who reported symptoms and never having previously attended a sexual health clinic were women, however we are keen to ensure that the experiences of men are represented in the data, therefore we aimed to achieve a sample of approximately equal numbers of women and men. Due to the well documented experience of difficulties recruiting men for social research studies [36], we recruited as many men into the sample as possible in the study time-frame.

Our sample was drawn from a representative national survey which increased the diversity of the sampling frame, an important feature of our approach. We used maximum variation sampling, a type of purposive sampling [37] to select participants with a diverse range of reported symptoms including different symptom types and multiple symptoms to generate appropriate data about their experiences (Table 1). Our sample consisted of those who had not previously attended a sexual health clinic as we know that past attendance can influence future care-seeking behaviour [38] and interpretations of current symptoms. We were also keen to include a variety of care-pathways and decision-making processes in our data. Sampling was iterative and responsive to feedback from earlier interviews and sampling procedures reviewed throughout the recruitment phase to ensure sufficient data was produced to answer the research questions ([39] p138). Natsal-3 participants who met our inclusion criteria were initially contacted by post then by telephone (or text message if there was no response from calling) to arrange the interview if they agreed to participate in an interview. Of 117 people we attempted to contact, 79 were uncontactable, 8 declined to take part, 2 did not meet study inclusion criteria and one dropped out before the interview took place. We recruited participants who had completed Natsal-3 most recently to help minimise attrition but included some participants from earlier waves of survey data collection to increase the variety of symptoms sampled.

The interview topic guide was piloted by FM on five participants January-February 2014. The full interviews were carried out by FM between May 2014 and March 2015 with 16 women and 11 men. Interviews took place at the participant’s home address (*n* = 24) or another convenient location specified by the participant (*n* = 3) and lasted between 35 and 108 min; the median length was 68 min. The interviews were structured around four main sections: STI perceptions; symptom meanings; care-seeking behaviour; STI stigma. The interview was flexible to the participant’s experiences and needs. We also embedded an interactive flash card activity within the interview to produce different types of data about STI perceptions. The activity involved comparative ranking of STIs from most to least (or similar end points) according to different themes: prevalence, infectiousness, visibility, severity, treatability and blameworthiness. Participants were asked to ‘think out loud’ and a photograph of the final flash card ordering was taken. The activity produced linked verbal and visual data and overcame some of the limitations associated with participants’ capabilities of spontaneously speaking about our research topic. We continued recruiting participants for interviews until the data produced was sufficient to answer our research questions and we had maximised the diversity of participants included in the study. Interviews were digitally recorded and transcribed verbatim by FM or a professional transcription company. Field notes were written to capture and reflect on the process of recruiting and interviewing participants and to inform subsequent analyses.

### Qualitative data analysis

All qualitative data are imported into NVivo V11 to facilitate organization of data in different formats (audio, written transcript, written field notes and photographs). We are drawing on principles of Interpretative Phenomenological Analysis [25, 40] with attention to discourse exploring the lived experiences and meanings of genito-urinary symptoms and decisions around care-seeking. This involves an idiographic approach, analysing transcripts individually, reading them through and recording initial notes, comments, questions, summaries, absences and uses of language through engagement with the data. These notes are then transformed into conceptual themes [41, 42] to capture the ‘essence’ of the data and these emergent themes are collated into groups to explore clustering and hierarchies of themes within the data. Organization and re-organization of these groups produces an overall coding framework composed of meta-themes, themes and sub-themes derived inductively from the data. The coding framework is then used to guide subsequent coding of other transcripts, ensuring data within each theme cohere meaningfully and themes encompass distinct concepts. We are remaining open to new codes emerging throughout the analysis process and are attentive to data that does not fit the coding framework or appears to differ from the rest of the dataset. Although we are taking a predominantly inductive approach, some *a priori* codes are developed from the topic guide and the emergent quantitative findings to facilitate cross-examination of the data. FM is coding all of the transcripts and approximately a third of the qualitative data are double coded by experienced qualitative researchers to ensure a comprehensive and rigorous analytical approach.

### Quality appraisal

We have considered both the Mixed Methods Appraisal Tool (MMAT) [43] and Good Reporting of a Mixed Methods Study (GRAMMS) [44] in the conceptualisation, development and description of this study. Reporting of results and outputs from this study will also follow this guidance.

### Integration of findings

This study uses quantitative survey data and qualitative data from semi-structured interviews. Integration of findings is the process through which enrichment and enhancement of each type of data occurs and greater understanding is achieved [45].

We will synthesise the data using multiple approaches for data integration [46]. The most prominent approach to integration will occur through the use of matrices. A ‘convergence coding matrix’ (ibid) (Table 2) will be used, adopted from the triangulation protocol [47] to present and then integrate findings from each strand of the mixed methods study, paying particular attention to areas of agreement, silence and dissonance [46]. Exploring the data in this way helps cut across the findings from each strand [47], highlight methodological discrepancy and increases the potential for gaining additional insights from using a mixed methods design.

**Table 2:** Example of a convergence coding matrix for integrating quantitative and qualitative findings

Research question	Quantitative findings	Qualitative findings	Integrated findings – agreement/ partial agreement/ silence/ dissonance
1. What are the social representations of STIs?			
2. How does stigma influence experiences of genito-urinary symptoms and care-seeking?			
3. How do people interpret genito-urinary symptoms?			
4. Why do some people with genito-urinary symptoms not seek care at sexual health clinics?			

As we have linked samples, we have both quantitative and qualitative data for a sub-set of participants. We are using the full Natsal-3 responses from each of the qualitative participants, if consent was given, to provide additional contextual analysis and create mixed method “cases” to illustrate our study and provide examples of specific symptom experiences and care-seeking pathways. As Natsal-3 contains 1792 variables (personal communication with Dr C. Mercer 4 June 2015), we will choose only those which can provide relevant additional data and focus on factors that are unlikely to change significantly over time, for example how old participants were when they first had sex or if they have previously been diagnosed with STIs. We collate quantitative and qualitative data on each participant in a ‘mixed methods matrix’ [46] (Table 3) to identify similarities and differences between data types for individual participants and look for patterns across individuals.

**Table 3:** Example of a mixed methods matrix for exploring patterns within and across individual participants

Participant ID number	Gender	Symptoms experienced past month	Symptoms experienced ever	Previous STI diagnoses	Suspected cause of symptoms	Hypothetical preferred service provider	Actual care-seeking for symptoms
1							
2							
3							
4							
Etc.…							

Throughout the integration phase, we are looking for areas of complementarity, divergence and ways to offset the weaknesses of each method with data from the other. We move backwards and forwards between the separate qualitative and quantitative datasets and integrated findings to identify if the reasons for patterns are methodological or empirical findings for this study.

## Discussion

### Summary

This is an ongoing mixed methods study exploring perceptions and social representations of STIs, genito-urinary symptoms and care-seeking behaviour in women and men in Britain. We are using data from Natsal-3 and follow-up semi-structured qualitative interviews with survey respondents to produce data from linked samples drawn from the population instead of recruited from medical settings. We will integrate the results from the quantitative and qualitative strands to produce synergistic findings that give richer and more meaningful insight than a single method approach.

### Strengths

Natsal-3 is a large survey of the British population with sufficient statistical power and a robust sampling strategy meaning that the quantitative results are broadly representative and therefore generalizable at a national level. This study will also provide the first estimates of the prevalence of genito-urinary symptoms in Britain and enables a unique approach to studying non-attendance as a facet of care-seeking behaviour. Our qualitative sample is diverse, maximising experiences of different symptoms in women and men and covering a wide range of ages and geographic locations. Semi-structured interviews enabled us to explore care-seeking more broadly than have been considered in Natsal-3, and to position decisions about needs and healthcare services in a specific social and cultural context.

A mixed methods approach enables us to use and integrate quantitative and qualitative data to study a complex social phenomenon, gain comprehensive insights into underlying mechanisms of experience and help explain quantitative results [17]. This would not be possible with a single method study. The linked datasets are a particular strength of this study as there is usually a trade-off between sample size and data linking in mixed methods research. We are able to make sense of population patterns within individual lived experiences and provide multi-dimensional insights into the research topic. We also have extensive information on the participants in our qualitative sample by using their full Natsal-3 responses. This gives us further opportunities to integrate data between quantitative and qualitative strands. The sequential study design enabled us to sample individuals with potential healthcare need, outside of medical settings. This is beneficial for our study and the opportunity it affords to study non-attendance behaviour but also contributes to a gap in the literature on healthcare services which is dominated by patient samples and research undertaken in clinical settings. The mixed methods design also broadens this study’s perspective of genito-urinary symptoms viewing them in other contexts besides STIs.

### Limitations

The time frames of the symptoms and care-seeking questions do not correlate as Natsal-3 asked about experience of symptoms in the past month and clinic attendance in the past year. We can deduce that individuals who hadn’t attended a clinic in the past year had also not been in the past month but do not have any quantitative data about care-seeking intentions or engaging with other health care providers, including GPs. Natsal-3 is a cross-sectional survey so it is not possible to determine causality of associations between symptoms and care-seeking. Survey data is self-reported (except for the urine and saliva sample testing) so there may be reporting bias, especially if the questions were perceived to be particularly sensitive. The time between quantitative and qualitative data collection ranged from 22 months to 44 months leading to high attrition of participants. It may not be possible to triangulate some of the quantitative and qualitative data because of the individual changes to behaviour and attitudes during this time.

### Other operational issues

All qualitative data collection was carried out by a white British female and this is likely to have an impact on the interview data as qualitative methods involve co-creation of data between the researcher and the interviewee ([39] p23-25). Some female participants in the qualitative sample mentioned that they would have been uncomfortable discussing their symptomatic experiences with a male interviewer and the reverse may have occurred for male participants. This phenomenon has been documented in other qualitative studies [48] and introduces additional analytic dimensions to the data produced.

### Application of findings

With increasing uncertainty in the provision of sexual healthcare services [49] and unknown population prevalence of symptoms, this study is timely in directly addressing gaps in the literature and answering questions relevant to public health and healthcare services. Our findings may produce new insights into lay decision-making about symptoms and healthcare-seeking behaviours by exploring individual explanatory frameworks for experiences. It may provide evidence for future commissioning of healthcare for genito-urinary health issues and inform the development of subsequent Natsal studies.

We feel that there is scope for adopting a similar approach using other national surveys to help explain quantitative patterns and trends, providing that the necessary permissions and protocols are put in place during the design and development phases to ensure ethical and data management issues are addressed appropriately.

## Abbreviations

CAPI, computer assisted personal interview; CASI, computer assisted self interview; GP, general practice/general practitioner; GRAMMS, Good Reporting of a Mixed Methods Study; GUM, Genito-Urinary Medicine; MMAT, Mixed Methods Appraisal Tool; Natsal, National Survey of Sexual Attitudes and Lifestyles; Natsal-3, Third National Survey of Sexual Attitudes and Lifestyles; NHS, National Health Service; STIs, sexually transmitted infections
